# The Turkish Bath

**Published:** 1896-04

**Authors:** Louis Lewis

**Affiliations:** Philadelphia


### SELECTIONS.

### The Turkish Bath.

BY LOUIS LEWIS, M.D., PHILADELPHIA.

Whoever indulges in a Turkish bath for the first time will be astounded at the vast amount of dead tissues and used-up bodily products that are expressed from his skin by the combined action of the hot air and massage. Countless rolls of effete material, that cannot be removed through the medium of ordinary perspiration, make their exit through the surface, by channels that were previously blocked and impervious. Simple perspiration—the first effect of the hot-air bath—acts on the smaller sweat-glands, and sets free the water, carbonic acid, and more or less nitrogen; but the larger sweat-glands, which are situated in the fatty tissues beneath the true skin, and contain the solid constituents lactates acetates, lime-salts, chloride of sodium, besides various extraneous matters present under unhealthy conditions, are only acted on by the addition of scientific hand-kneading. The retention of these extraneous matters, urea, uric acid, acetone, bile-products, etc., leads to toxemic headaches, erythematous swellings, rheumatism, gout and jaundice. Another system of glands, the sebaceous or oil-glands, are also apt to become blocked, and their imprisoned tallowy contents—olein, stearin, palmitin—give rise to unsightly varieties of acne on the face, chest and shoulders. But these glands are nearer the surface than the sweat-glands, and thus are more readily relieved of their contents. The sweat-glands also contain some fatty matter, similar to, but not identical with, the contents of the sebaceous glands. The exit of sweat results partly from increased blood-supply, but more from reflex action in the sweat-centers, kindled through the medium of the nerve fibres.

No sensible person can doubt the expediency of ousting all these intruders from the system by a method so agreeable and effectual as the Turkish bath. Then comes a sensation of relief from some indefinable general obstruction, as though one were actually breathing through every portion of the body, and a feeling of buoyancy only comparable to "walking on air." A little muscular languor may remain, which, however, is not fatigue, yet invites one to indolent repose on a couch provided for the purpose; and after a short dreamy period, devoted to "thinking of nothing at all" (like the jolly young waterman), one rises as a "giant refreshed."

Numerous minor ailments and functional disorders are removed or ameliorated by the Turkish bath. The expectoration, hoarseness and oppression of breathing, attendant on "taking cold," are speedily relieved, also the lassitude and aching of limbs. In febrile conditions, it is necessary to restore the balance between oxidation and nutrition, or, in other words, between the production of heat and its consumption; here the bath affords a rational remedy, by reducing and regulating the temperature. Heat is a source of motion in the animal body, and must not be unnecessarily wasted. Residents of cities, who lead sedentary lives, and take insufficient exercise in proportion to a free mode of living, are liable to a general plethoric condition, marked by a coated tongue, florid face, suffused eyes and bounding pulse; or the blood itself may be tainted with retained excrementitious material, as signalized by a moist tongue, pallid face, sallow skin and depraved secretions. A regular use of the Turkish bath will go far to control the blood-supply, and, by removing the offending matter, restore the various organs to their normal state. The bath is very useful in neurasthenia, with a dry tongue, wayward appetite, disordered vision, tinnitus aurium, and headache; and in vertigo, even when the dizziness affects the gait and causes a dread of walking alone. The bath tranquilizes the vital condition of all the nervous centers. Many persons are worried by morbid "thick-coming fancies" that trouble the brain, and weigh upon the heart; these are commonly called "a fit of the blues" or "the vapors," and they are quickly exorcised by the Turkish bath. Chronic sleeplessness, whether due to rheumatic, gouty or dyspeptic disorders, yields to a course of baths, through the depurative effect of free transpiration and the functional activity that is awakened by the massage. Headaches of nervous and congestive origin, and those due to the toxemic influence of urea, bile, alcohol and malaria, succumb to this agent, when timely employed. Spinal irritation, rheumatism, lumbago and sciatica, asthma, chronic bronchitis and winter cough, irregular work of the kidneys and liver, are all alleviated by hot air and massage. Eczema, psoriasis, urticaria, prurigo and other skin diseases are benefited; but for obvious reasons these are unsuited for treatment in public establishments. In fine, in any of the other conditions alluded to and in many functional disturbances outside of organic change, I would say to the patient, without discrimination, seriously and literally, "Go to bath," and let it be a Turkish one.—*The Times and Register.*

